# Bicarbonate is a key regulator but not a substrate for O_2_ evolution in Photosystem II

**DOI:** 10.1007/s11120-024-01111-8

**Published:** 2024-07-22

**Authors:** David J. Vinyard, Govindjee Govindjee

**Affiliations:** 1 Department of Biological Sciences, Louisiana State University, Baton Rouge, LA 70803 USA; 2 Department of Biochemistry, Department of Plant Biology, Center for Biophysics and Quantitative Biology, University of Illinois at Urbana-Champaign, Urbana, IL 61801 USA

**Keywords:** Photosystem II, Water oxidation, O_2_ evolution, Bicarbonate

## Abstract

Photosystem II (PSII) uses light energy to oxidize water and to reduce plastoquinone in the photosynthetic electron transport chain. O_2_ is produced as a byproduct. While most members of the PSII research community agree that O_2_ originates from water molecules, alternative hypotheses involving bicarbonate persist in the literature. In this perspective, we provide an overview of the important roles of bicarbonate in regulating PSII activity and assembly. Further, we emphasize that biochemistry, spectroscopy, and structural biology experiments have all failed to detect bicarbonate near the active site of O_2_ evolution. While thermodynamic arguments for oxygen-centered bicarbonate oxidation are valid, the claim that bicarbonate is a substrate for photosynthetic O_2_ evolution is challenged.

## Introduction

Oxygenic photosynthesis is initiated by light-driven water oxidation and plastoquinone (PQ) reduction in the Photosystem II (PSII) reaction center. On the electron donor side of PSII, water is split and O_2_ is formed at a Mn_4_CaO_x_ active site known as the oxygen-evolving complex (OEC). The protons stripped from water are channeled to the thylakoid lumen. On the electron acceptor side of PSII, electrons are transferred from the primary PQ acceptor, Q_A_, to the secondary PQ acceptor, Q_B_, via a non-heme iron (NHI). Protons from the thylakoid stroma are consumed upon Q_B_ reduction. The net reaction of PSII is summarized as

$$ \begin{array}{l}{\rm{2}}{{\rm{H}}_{\rm{2}}}{\rm{O + 2PQ + 4}}{{\rm{H}}^{\rm{ + }}}_{{\rm{stroma}}}{\rm{ + }} \ge {\rm{4h}}\upsilon \\\to {{\rm{O}}_{\rm{2}}}{\rm{ + 2PQ}}{{\rm{H}}_{\rm{2}}}{\rm{ + 4}}{{\rm{H}}^{\rm{ + }}}_{{\rm{lumen}}}{\rm{.}}\end{array} $$

The PSII electron donor side operates using cofactors with very positive reduction potentials. Electrons are transferred from water (E_m_ (O_2_/H_2_O) = + 0.88 V at pH 6) to the OEC (E_m_ (S_*n*+1_/S_n_) = + 1.1 V) to tyrosine Z (E_m_ (Y_Z_^•^/Y_Z_) and finally to the hole (positive charge) in the ground state of the primary chlorophyll electron donor, P_680_ (E_m_ (P_680_^+^/P_680_) = + 1.25 V) (reviewed in (Vinyard et al. 2013; Blankenship 2021)) These values are remarkably positive in all of biochemistry and have led to PSII being the only enzyme capable of oxidizing water to O_2_.

Studies in the early 1940s showed that the isotopic composition of oxygen was nearly identical in water and photosynthetically produced O_2_, and concluded that water is the source of O_2_ in photosynthesis (Ruben et al. 1941; Vinogradow and Teis 1941). We acknowledge that these systems are complicated and could be affected by spontaneous or biologically-driven isotope mixing (for example, see discussions in (Metzner 1975). The source of O_2_ was later challenged when Otto Warburg and G. Krippahl observed the requirement of bicarbonate in the photosynthetic light reactions (Warburg and Krippahl 1958, 1960). Comprehensive reviews about the role(s) of bicarbonate in PSII have been published (van Rensen and Klimov 2005; McConnell et al. 2012; Shevela et al. 2012; Swain et al. 2023).

Based on reasoning provided in this perspective, a consensus has now been reached among PSII researchers that O_2_ originates solely from water (for reviews, see (Vinyard and Brudvig 2017; Lubitz et al. 2019; Shevela et al. 2023). However, alternative hypotheses usually involving bicarbonate that were once dominant (see discussion in (Warburg 1964), have persisted (for example, (Stemler 1980; Castelfranco et al. 2007), and are making a resurgence (see for example, (Wu 2021, 2023; Kelath Murali et al. 2022; Guo et al. 2024)). In general, these recent reports argue that bicarbonate is thermodynamically easier to oxidize than water, and that isotope tracking experiments have been misinterpreted because PSII facilitates isotope mixing.

Here, we reason that bicarbonate is indeed very important for PSII function but does not serve as a substrate for O_2_ evolution. In addition, we reevaluate the thermodynamics of oxygen-centered bicarbonate oxidation relative to direct water oxidation.

## Bicarbonate regulates PQ reduction

**Fig. 1:**
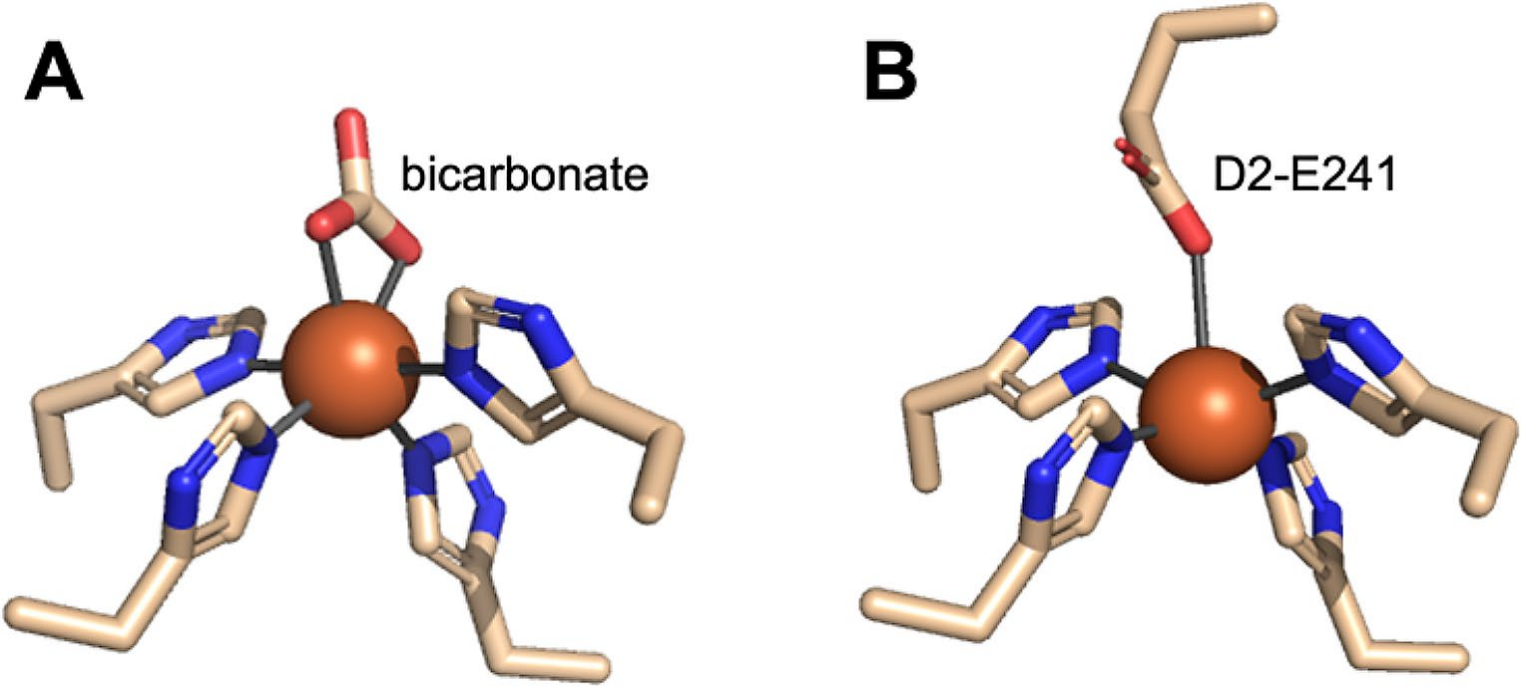
In mature PSII (**A**), the NHI (orange sphere) is ligated by a bicarbonate ion (Umena et al. 2011). In a PSII assembly intermediate (**B**), the NHI is ligated by a glutamate side chain (Zabret et al. 2021). Image generated in PyMOL from PDB IDs 3WU2 and 7NHQ

In PSII, bicarbonate acts on the electron acceptor side (Wydrzynski and Govindjee 1975) by ligating the NHI cofactor that resides between Q_A_ and Q_B_ (Xiong et al. 1996). This NHI is additionally ligated by four histidine residues from the D1 and D2 subunits of PSII (Fig. 1A) (Umena et al. 2011). In this configuration, the NHI promotes efficient electron transfer from Q_A_^−^ to Q_B_ (reviewed in (Müh and Zouni 2013). The bicarbonate ligand can be removed completely or substituted by formate or acetate, which inhibits electron transfer to Q_B_ (see (Shevela et al. 2012). In some PSII assembly intermediates, the NHI is ligated by a carboxylate from a D2 glutamate residue (Fig. 1B) (Zabret et al. 2021; Gisriel et al. 2022a) that is presumably replaced with bicarbonate at a later stage. When bicarbonate is absent, O_2_ acts as an electron acceptor (Ananyev et al. 2018) resulting in superoxide formation on the PSII electron acceptor side (Fantuzzi et al. 2022) and charge recombination between P_680_^+^ and Q_A_^−^ becomes more favorable (Brinkert et al. 2016). These pathways are photoprotective because they minimize the formation of triplet chlorophyll as ^3^P_680_ which decays by reacting with O_2_ to form ^1^O_2_ (Vass and Cser 2009). This reactive oxygen species damages biomolecules including those involved in photosynthesis (Triantaphylidès et al. 2008).

A simple yet elegant model is now available in which systems with adequate CO_2_/HCO_3_^−^ are likely to have the PSII NHI ligated by bicarbonate and have the capacity for productive photosynthesis (Brinkert et al. 2016; Tikhonov et al. 2018; Fantuzzi et al. 2022). In this model, the flux of PQ reduction by PSII is maximized and, in downstream steps in the Calvin-Benson Cycle, CO_2_ is fixed into carbohydrates. Systems without adequate CO_2_/HCO_3_^−^ could have the PSII NHI in an alternative ligation environment thus changing its reduction potential and drastically decreasing reactions leading to net photosynthesis. In the absence of bicarbonate, PQ reduction by PSII would be minimized and the excess energy would be dissipated as heat through charge recombination. We note that this model was established using in vitro experimental data and has yet to be confirmed in vivo.

## Bicarbonate can facilitate proton release during water oxidation

Splitting water and producing O_2_ is accompanied by a release of protons. The OEC is connected to the thylakoid lumen via hydrogen-bond networks that can act as proton channels (Vogt et al. 2015). These networks are interconnected with each other (Kaur et al. 2021), but there are differences in these networks in different organisms (Gisriel et al. 2022b). Further, chloride ions are present in all high resolution PSII structures (see, for example, (Umena et al. 2011) and are used to tune the pK_a_ values of the side chains of nearby components, as well as water molecules in these hydrogen-bond networks to promote efficient proton transfer from the OEC to the thylakoid lumen (Pokhrel et al. 2011). Bicarbonate has been demonstrated to act as a mobile proton carrier between the OEC and the lumen (Koroidov et al. 2014). This role of bicarbonate has been shown to enhance PSII activity under high light conditions (Koroidov et al. 2014), in hydrogen-bond network point mutants (Ananyev et al. 2005; Banerjee et al. 2019), and in *Arthrospira maxima*, a cyanobacterium from a hyper-carbonate environment (Carrieri et al. 2007).

## PSII is capable of oxidizing substrates other than water

Given the very positive reduction potentials of PSII electron donor side cofactors (+ 1.1–1.25 V, see above), it is reasonable that this enzyme can also oxidize other molecules besides water. However, the OEC binding site is well protected from the outside environment by reaction center subunits and extrinsic subunits that protect and stabilize the OEC (reviewed in (Bricker et al. 2012).

### Mn^2+^ is oxidized during OEC photo-assembly

The OEC is assembled in situ from free Mn^2+^, Ca^2+^, and water (reviewed in (Oliver et al. 2022). During this process, the electron donor side of PSII oxidizes Mn^2+^ ions to Mn^3+^ or Mn^4+^ ions. Simultaneously, water is deprotonated to form μ-(hydr)oxo ligands. In vitro, apo-OEC PSII has been observed to hyperaccumulate higher valent Mn ions when Ca^2+^ concentrations are low (Chen et al. 1995) or under high illumination intensities (Chernev et al. 2020). These results, as well as some others, have been used to propose an evolutionary mechanism in which an ancestral type II reaction center used Mn^2+^ ions as its electron donor instead of water (Zubay 2000; Dismukes et al. 2001; Allen and Martin 2007; Fischer et al. 2015; Chernev et al. 2020).

The early steps of OEC assembly have been known to be highly inefficient (Radmer and Cheniae 1971) and require multiple deprotonation events. Charles Dismukes and coworkers have shown that bicarbonate not only makes OEC assembly faster (Baranov et al. 2000), but also makes the process more efficient (Baranov et al. 2004). This effect is likely to involve two things. First, bicarbonate-ligated Mn^2+^ is thermodynamically easier to oxidize to Mn^3+^ than [Mn(H_2_O)_6_]^2+^ (Dismukes et al. 2001). Second, proton release controls the rate-determining step of OEC assembly (Vinyard et al. 2019) and bicarbonate may serve as a mobile proton carrier to facilitate this step. In addition, chloride also enhances OEC assembly (Miyao and Inoue 1991) using similar mechanisms as bicarbonate. First, chloride makes bound Mn^2+^ easier to be oxidized (Russell and Vinyard 2022). Second, chloride promotes efficient proton release (Vinyard et al. 2019). Further research is needed to disentangle bicarbonate vs. chloride effects during OEC assembly.

### Small molecules can access the OEC and be oxidized

Several small molecules other than water can donate electrons to the OEC. For example, hydroxylamine (Bouges 1971; Cheniae and Martin 1972), hydrazine (Kok and Velthuys 1977), and hydrogen peroxide (Velthuys and Kok 1978) can reduce Mn^3+/4+^ ions in the OEC. Access of these molecules to the OEC is increased when extrinsic subunits are depleted (Ghanotakis et al. 1984). Similar to an evolutionary mechanism involving Mn^2+^ described above, hydrogen peroxide has also been proposed to have been a donor in a PSII ancestral reaction center (Blankenship and Hartman 1998).

Recently, Gary Brudvig and coworkers showed that ammonia and iodide can be oxidized by the OEC in mutants with poor chloride binding (Shin et al. 2024). They propose that that in addition to chloride’s role in promoting proton release, it also enhances OEC substrate selectivity in favor of water. As an extension of this concept, we hypothesize that chloride also limits access of bicarbonate to the OEC. If this concept is experimentally validated, bicarbonate would be limited to hydrogen bond networks through which protons are shuttled to the lumen.

### Oxidizing hydrated CO_2_ is thermodynamically feasible

**Fig. 2:**
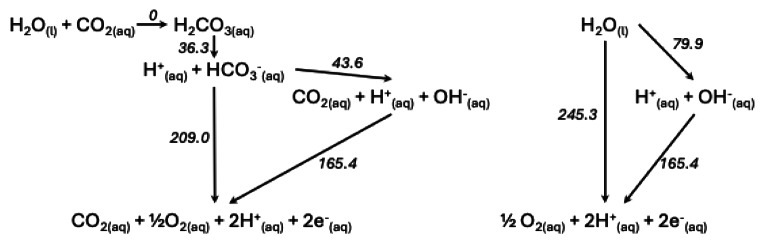
Calculated Δ_r_G° values in kJ mol^− 1^ under chemical standard conditions (298.15 K, 1 bar, 1 M concentration of each aqueous species). Data are compiled from National Bureau of Standards tables (Wagman 1982) with a correction for Δ_f_G° for the dissociation of H_2_CO_3(aq)_ from (Alberty 1997). This scheme is based on an earlier analysis by (Dismukes et al. 2001)

Suggestions that bicarbonate is a substrate for O_2_ production are thermodynamically reasonable. In Fig. 2, an updated version of a thermodynamic analysis by (Dismukes et al. 2001) is presented. This analysis is performed using chemical standard conditions at equilibrium and caution is warranted in applying it to conditions in vivo.

On the left scheme in Fig. 2, CO_2(aq)_ is first hydrated to H_2_CO_3(aq)_, which then can dissociate to H^+^_(aq)_ and HCO_3_^−^_(aq)_. These combined reactions have an *effective* pK_a_ of approximately 6.1. Therefore, in the thylakoid lumen at pH 6, CO_2(aq)_ and HCO_3_^−^_(aq)_ are present in similar concentrations. H^+^_(aq)_ + HCO_3_^−^_(aq)_ can be oxidized to form O_2_ with a standard free energy input of 209.0 kJ mol^− 1^. (Note that the carbon atom is already fully oxidized in bicarbonate and the oxidation is oxygen centered.) This energy gap can be broken into two steps by dissociating HCO_3_^−^_(aq)_ to CO_2(aq)_ and OH^−^_(aq)_ and then performing the oxidation reaction to form O_2_.

### Oxidizing water directly is also thermodynamically feasible

On the right scheme in Fig. 2, water is directly oxidized to O_2_ with a standard free energy input of 245.3 kJ mol^− 1^. This energy gap can also be broken into two steps by first dissociating water to H^+^_(aq)_ and OH^−^_(aq)_ and then performing the oxidation reaction to form O_2_.

The above analysis shows that oxidizing bicarbonate under chemical standard conditions (pH 0) requires only 209.0 kJ mol^− 1^ of free energy input, while oxidizing water directly requires 245.3 kJ mol^− 1^ of free energy input. When scaled to pH 6 (typical of the lumen), oxidation of bicarbonate requires a 20% lower free energy input than direct oxidation of water (140.7 kJ mol^− 1^ vs. 176.8 kJ mol^− 1^, respectively). These values correspond to + 0.7 V for bicarbonate and + 0.9 V for water. Given that the reduction potential of the OEC (S_*n*+1_/S_n_) is + 1.1 V, the OEC is thermodynamically capable of oxidizing either species.

## The PSII electron donor side has not been shown to have any high affinity bicarbonate binding sites

Bicarbonate has long been postulated to function near the OEC (Stemler and Govindjee 1973). However, high-resolution structural studies using cryogenic electron microscopy (for example, (Kato et al. 2021; Gisriel et al. 2022b; Hussein et al. 2024), synchrotron X-ray sources (for example, (Guskov et al. 2009; Umena et al. 2011), and X-ray free electron laser sources (for example, (Kupitz et al. 2014; Suga et al. 2015, 2017; Young et al. 2016) have failed to detect bicarbonate bound near the OEC. Although an early 3.5 Å study suggested a bicarbonate near the OEC (Ferreira et al. 2004), it has not been observed in that location in later structures at higher resolutions. Further, a spectroscopic study has failed to detect any high affinity bicarbonate on the electron donor side (Aoyama et al. 2008). Efforts to quantify the number of bicarbonate ions bound per PSII reaction center have consistently produced values of nearly one (Govindjee et al. 1991, 1997; Tikhonov et al. 2018) or less than one (Shevela et al. 2008; Ulas et al. 2008). These observed values are inconsistent with any additional bicarbonate binding sites besides that on the electron acceptor side of PSII. However, mobile bicarbonate ions may function to move protons on the electron donor side, as noted in this perspective.

## O_2_ originates from water

Bicarbonate is a key cofactor of PSII. Its role on the PSII electron acceptor side is critical. Its supporting roles in facilitating proton release and OEC assembly help PSII optimize its function. If bicarbonate was bound near the OEC it would be easier to oxidize than water. However, bicarbonate simply has not been observed near the OEC using structural, spectroscopic, or biochemical approaches. Instead, the OEC is surrounded by water molecules which it is thermodynamically capable of oxidizing. O_2_ produced by PSII originates only from water.

## Data Availability

No datasets were generated or analysed during the current study.

## Abbreviations

E_m_: Reduction potential
NHI: Non–heme iron
OEC: Oxygen–evolving complex
P_680_: Primary chlorophyll–a electron donor of Photosystem II
PSII: Photosystem II
PQ: Plastoquinone
Q_A_: Primary plastoquinone electron acceptor
Q_B_: Secondary plastoquinone electron acceptor
S_n_: Storage state of the OEC
Y_Z_: Tyrosine Z
